# Public Policies on Obesity: A Literature Review of Global Challenges and Response Strategies

**DOI:** 10.7759/cureus.62758

**Published:** 2024-06-20

**Authors:** Xiaoyue Lan, Norhasmah Sulaiman

**Affiliations:** 1 Department of Nutrition, Universiti Putra Malaysia, Serdang, MYS

**Keywords:** social determinants of health, health promotion, health politics, global nutrition, nutrition status, obesity policy

## Abstract

As a complex and multifactorial health problem, obesity results from the interaction of genetic, environmental, dietary, and lifestyle factors. Globally, the increase in obesity and related chronic diseases has been associated with global trade liberalization, rapid urbanization, and economic growth. This article is a narrative literature review on the global obesity problem and explores the global challenges of obesity and strategies to address them. The research methodology included a retrieval of peer-reviewed articles, including PubMed, ScienceDirect, and Google Scholar. Specific search terms like “obesity”, “policy”, “nutrition”, and “global”, outline the impact of obesity on global health and social systems, as well as policy effectiveness and gaps that exist. The outcome reveals regional differences in obesity rates and provides an analysis of the policies that countries have implemented to address obesity and their effectiveness, in particular concerning improving the quality of diets and limiting the intake of added sugars. Despite some policies proving effective, the challenge of obesity is far from being fully addressed, necessitating robust international efforts and strategies.

## Introduction and background

**Figure 1:**
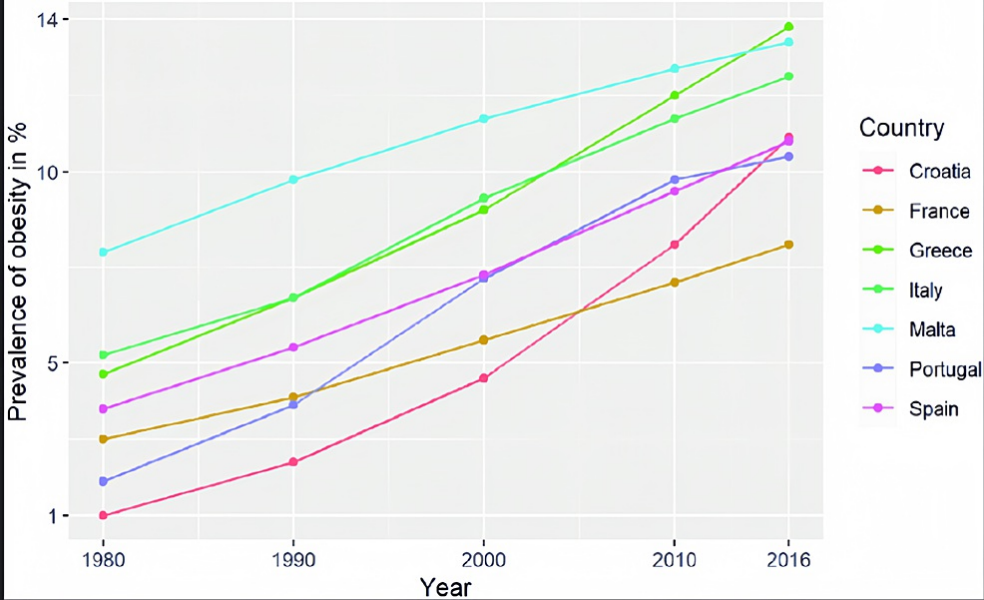
Global obesity growth chart from 1980 to 2016 Source: Epidemiology of Obesity in Children and Adolescents; Nittari et al., 2020 (https://www.researchgate.net/publication/344310582_Epidemiology_of_Obesity_in_Children_and_Adolescents). Used under CC BY 3.0.

Obesity is a complex and multifactorial health issue affecting individuals worldwide. It is a chronic metabolic disease that results from the interaction of genetic, environmental, dietary, and lifestyle factors, etc. [1]. The World Health Organization (WHO) defines body mass index (BMI) over 25 as considered overweight and over 30 as obese [2]. Excessive adiposity is an important risk factor for morbidity and mortality from type 2 diabetes mellitus (T2DM), cardiovascular diseases, and some cancers. The worldwide increase in overweight, obesity, and related chronic diseases has largely been driven by global trade liberalization, rapid urbanization, and economic growth [3]. From a historical point of view, a trend toward overweight and obesity in higher-income countries, such as the United Kingdom, United States, Switzerland, Canada, etc., is a major increase seen in the 1980s to the present. In contrast, minimal obesity in most of the developing and transitional world until the late 1980s. This shift is due to similar forces that have driven the obesity trend in higher-income countries, including changes in diet, activity levels, and the influence of Western overeating culture. The affected groups in these countries are also starting to mirror those in higher-income countries, with more vulnerable and lower socioeconomic status populations and individuals with lower educational status being more affected [3]. There have been rapid changes in the past 20 years [4]. Figure 1 shows the global obesity growth chart from 1980 to 2016.

The WHO and World Obesity Federation regularly compile data on obesity rates worldwide. The WHO publishes an annual report called the World Health Statistics, which includes the most recent available data on health and health-related indicators. The World Obesity Federation publishes the World Obesity Atlas annually, which includes global, regional, and national estimates for the prevalence of obesity up to 2035. According to the World Population Review, in 2021, the global prevalence of obesity was estimated to be around 13.3% [5]. This percentage varied significantly by region and country. I randomly selected a few countries to explain the situation, including developed and developing countries.

**Figure 2:**
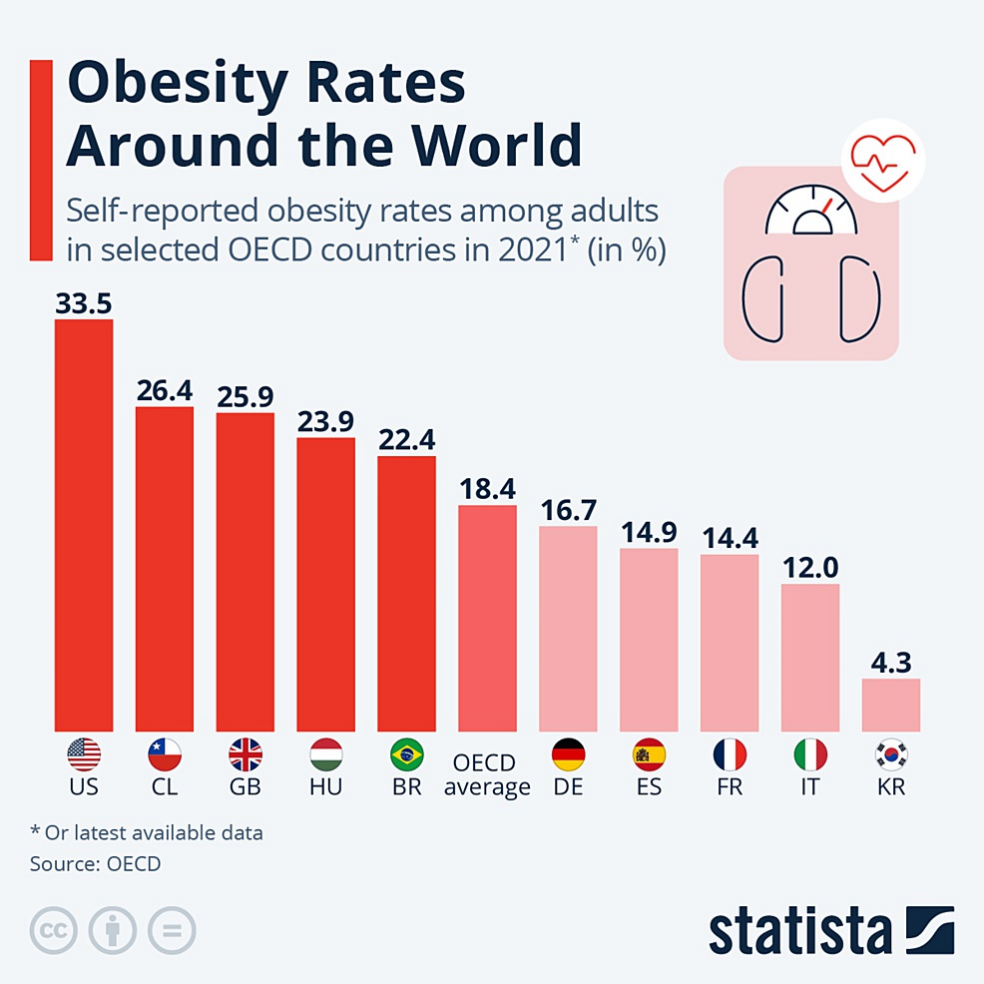
Obesity rates around the world in 2021 Source: Statista (https://www.statista.com/chart/20057/obesity-rates-eu/)

According to data from the Centers for Disease Control and Prevention (CDC), in 2019-2020, the prevalence of obesity among adults was around 42.4% and the United States has consistently reported high rates of obesity [6]. According to the World Population Review, the obesity rate in Mexico was estimated to be over 28% in 2021 and Mexico has also faced a significant obesity challenge (Figure 2) [7]. According to the NHS Digital Health Survey for England, in 2019, around 28.0% of adults in England were classified as obese, and the United Kingdom has experienced a gradual increase in obesity rates [8]. China has seen a rise in obesity rates, particularly in urban areas. According to a study published in The Lancet in 2019, the prevalence of obesity in China more than tripled between 1990 and 2019, reaching 16.3% [9]. India has traditionally had lower obesity rates compared to some Western countries, but there has been an upward trend. According to the National Family Health Survey (NFHS-4) conducted in 2015-16, the prevalence of obesity among women (15-49 years) was 20.7%, and among men (15-49 years), it was 18.9% [10]. Brazil has also seen an increase in obesity rates. According to the Brazilian Institute of Geography and Statistics (IBGE), in 2019, approximately 20.3% of the Brazilian population aged 18 years and older were classified as obese [11].

**Figure 3:**
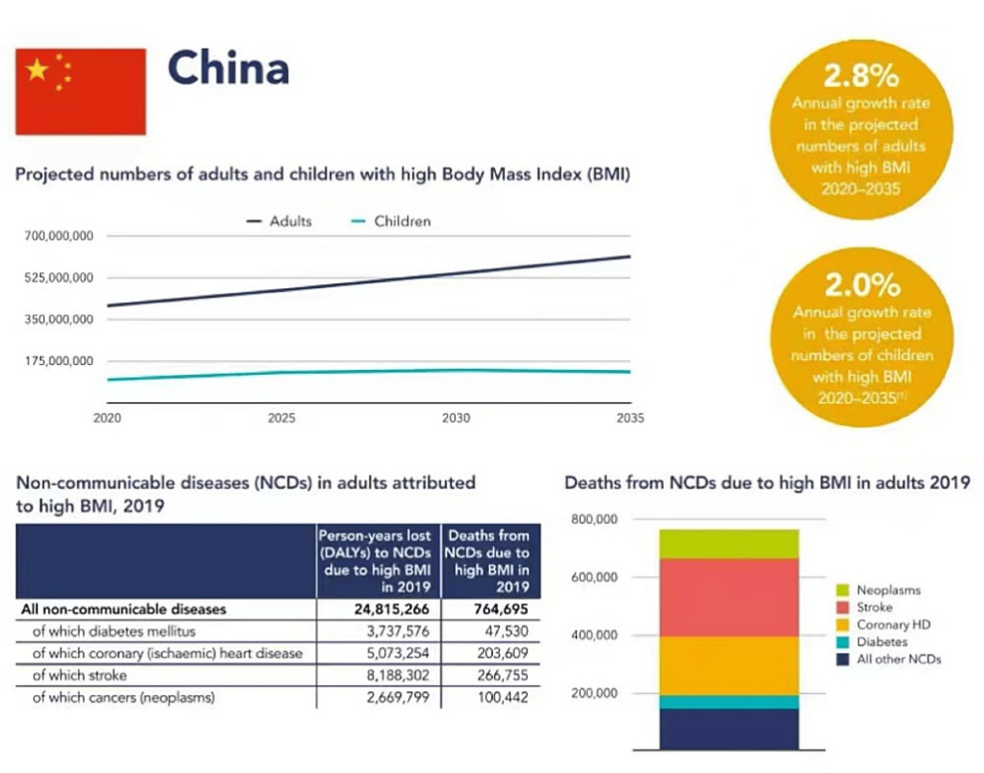
BMI prediction, obesity, and non-communicable disease relationships in China Source: https://zhuanlan.zhihu.com/p/641120242

The issue has grown to epidemic proportions, with over 4 million people dying each year as a result of being overweight or obese according to the global burden of disease from WHO in 2017 [12]. In the case of China, it's a graphic view of the relationship between BMI prediction and non-communicable diseases (NCDs) for better visualization of the dangers of obesity (Figure 3).

The reason why there has been such a shift is that the nutritional transition in low- and middle-income countries is typically characterized by increased consumption of animal fats and proteins, refined grains, and added sugars [13]. So now, high income and obesity are no longer necessarily linked. Modern life is much more convenient and faster, mobile phones and computers are used frequently, people spend more time sitting in front of screens than before, and a sedentary lifestyle has become a major factor in obesity [14]. Countries have contributed to the development of obesity policies, and it remains worth discussing what the implemented obesity policies have achieved in practice, which strategies have proved successful, and which have challenges and shortcomings.

## Review

Methodology

The review employed a comprehensive research approach, including peer-reviewed articles, research studies, and authoritative reports. This literature review used electronic databases (PubMed, ScienceDirect, and Google Scholar) to search for articles on overweight and obesity policy settings worldwide from 2008 to 2023 based on keywords “obesity”, “policy”, “nutrition”, and “global”. It is designed to ensure the inclusion of high-quality, appropriate, and evidence-based literature. The inclusion criteria for the search included case reports and original articles. Language restrictions were applied. For example, articles in English were selected. The selection of articles was based on inclusion criteria and search terms, and the titles and abstracts of all resources were screened independently. Selected titles and abstracts were then screened to check whether their content was likely to answer the review questions such as the impact of obesity policies. This included the impact of obesity on global health and social systems, linkages with chronic disease, health care costs, and quality of life, as well as issues of policy effectiveness and gaps and challenges. Irrelevant abstracts were excluded and the full articles with the selected abstracts were then retrieved. Thirty-eight articles were retrieved.

Discussion

Obesity, a major concern among NCDs worldwide, has seen a surge in prevalence. Over the past decade, numerous countries have initiated efforts to curb this growing health issue. This review mainly covers obesity policies in eight countries: China, the United States, the United Kingdom, Canada, India, Chile, Mexico, and Brazil. Over the past decade, in response to the increasing burden of obesity and related chronic diseases, many countries have adopted policies aimed at improving the quality of their diets, with a focus on limiting the intake of sugar-sweetened beverages (SSBs), which are the largest source of added sugars in many populations [15]. The WHO guidelines and Dietary Guidelines for Americans (DGA) recommend limiting the intake of added sugars to no more than 10% of caloric intake [16]. DGA is authored by the U.S. Department of Agriculture (USDA) and the U.S. Department of Health and Human Services (HHS). The Centers for Disease Control and Prevention (CDC) also uses these guidelines as a basis for its nutrition policies and programs. So, in that sense, the CDC does support the Dietary Guidelines for Americans. So far, several countries such as Mexico, South Africa, and the United Kingdom, have implemented taxes on SSB as a strategy to reduce intake and generate revenue to support public health efforts [17]. However, the impact of these taxes is usually observed over a period of years. Evidence shows that implementing taxes on SSBs increases product prices and reduces demand, resulting in fewer purchases [17].

Sales of sugar-sweetened beverages have declined since Mexico began to implement its sugar tax policy. The Mexican government has imposed a tax of 1 Mexican peso per liter of soda since 2014 and a 5% tax on snacks [18]. In the first year of the implementation of the Mexican Sugar Tax, sales of sugar-sweetened beverages in Mexico fell by 5.5%, and by 9.7% the following year, according to the National Institute of Statistics and Geographic Information of Mexico (INEGI) [18]. Childhood obesity rates fell. In Mexico, despite the decline in childhood obesity rates, the situation remains a significant public health concern. Overweight and obesity in school-age children is a growing problem with serious repercussions for future life. New strategies are needed that focus on involving food systems, which translates to healthy and sustainable diets. This policy has been highly successful in Mexico. Brazil, also a Latin American country, has been successful in reducing obesity rates with its sugar tax policy [18]. Likewise, in Canada, the Government has improved school meals and strengthened food labeling regulations to make it easier for consumers to understand the nutritional value of products [19]. In addition, the Canadian Government has strengthened the regulation of food advertisements, especially those aimed at children, to promote healthy eating habits, which is why Canada's obesity policy is also successful [19].

However, some policies are not very successful. The primary measure of success is a decrease in the prevalence of obesity in the population targeted by the policy. Success can also be measured consumption of sugar-sweetened beverages or increased physical activity. The sugar tax imposed by the Government of Chile on sugary beverages is 0.02% of its Gross Domestic Product (GDP), which is the highest among Latin American countries [20]. Studies have shown that sales of solid beverages have decreased while sales of untaxed beverages, such as water, have increased in Latin America [21]. However, the policy may result in making sugary foods more unaffordable for the poor, thereby increasing their financial burden. Therefore, the sugar tax policy may make it more difficult for them to access nutrition. The implementation of sugar tax policies requires strict regulatory and punitive mechanisms to ensure their successful implementation. However, given the complexity of Chile's administrative system and enforcement environment, policy implementation may face greater challenges. The sugar tax policy may have certain impacts on related industries such as the sugar-sweetened beverage manufacturing and food processing industries. This may lead to problems such as business closures and rising unemployment, which will hurt the economy in Chile. In addition, in the United Kingdom, a sugar tax led to a reduction in the sugar content of reformulated beverages [22]. Whether these early benefits of SSB taxes continue and translate into health improvements will be important to monitor. However, consumers may choose alternative high-sugar beverages or foods instead of reducing their overall sugar intake. This substitution effect may reduce the overall effectiveness of the policy, as people may choose to buy other unhealthy products that are not affected by the sugar tax.

The United States has implemented the Food Pyramid and MyPlate educational programs to promote a balanced diet and reduce obesity [23]. Although the United States has made efforts such as Public Health Interventions, Culturally Appropriate Programs, and State of Obesity Reports to address the problem of obesity, the effects of these policies are not obvious. This is due to the impact of supermarket promotions and convenience foods on the obesity problem. In the United States, low-quality, high-calorie foods (e.g., fast food, fried, puffed foods, etc.) are readily available while foods with a high percentage of protein are unaffordable. Cheap and high-calorie foods have become an important part of the American food culture. Highly processed foods have the same negative health effects. The fact that obesity is unevenly distributed among racial groups in the U.S. is a shortcoming in the U.S. obesity policy; in other words, the uneven distribution of obesity among racial groups in the U.S. highlights the need for more inclusive and equitable obesity policies that effectively address the needs of all populations [23]. Similarly, in China, the government promotes dietary guidelines and strengthens school food safety regulations, but over-reliance on foods high in sugar, salt, and oil still exists in some areas. According to the CDC, the prevalence of obesity among Chinese adults more than doubled between 2004 and 2018 (from 3.1% to 8.1%) [9]. This indicates that although China has adopted several policies to prevent and control obesity, the effectiveness of these policies is not obvious. As many as 135 million people are overweight or obese in India, according to a 2016 statistic; this number is still increasing rapidly, and India's overweight population is surpassing its undernourished population [24]. India has implemented several obesity programs and policies, such as promoting healthier eating habits by providing incentives and support for the production and marketing of healthier food products, including providing incentives for producers to reduce added sugars and fats [24]. However, persistent social inequalities and gaps between rich and poor, as well as inadequate infrastructure in some areas, affect the implementation of policies in India [24].

While we have seen some important steps aimed at reversing the global obesity epidemic, the unrelenting growth suggests that our efforts are not enough [25]. In the coming years, as the world grapples with the rising burden of obesity and chronic disease alongside the epidemic, there will be an urgent need for coordinated action across all countries/regions and sectors of society to prioritize the reduction of obesity risk factors to ensure the health and well-being of the global population. Considering factors such as health behaviors, conditions and medications, and genetics, and fostering multi-sectoral collaborations can help policymakers develop comprehensive, evidence-based, and contextually relevant obesity policies that address the complex nature of the issue.

These are some factors affecting obesity policy-making: Cultural norms, and societal attitudes toward food, body image, and health can influence obesity policy-making. Policies need to consider cultural and social contexts to ensure their relevance and effectiveness, approaches that respect diverse cultural backgrounds and address social determinants of health are more likely to be successful [26]. Also, global and regional collaborations can influence obesity policy-making by facilitating the exchange of best practices, sharing experiences, and coordinating efforts [25]. International organizations, such as the WHO, play a vital role in setting global agendas, providing guidance, and supporting countries in developing obesity policies. In addition, the availability and analysis of epidemiological data on obesity prevalence, trends, and associated health outcomes play a crucial role in shaping obesity policies, this data helps policymakers understand the scale of the problem, identify high-risk groups, and evaluate the effectiveness of interventions [26]. Last but not least, strong political will and leadership are essential for prioritizing obesity as a public health issue and driving policy action. Political support can help overcome resistance from various stakeholders and ensure the allocation of resources for obesity prevention and management efforts. Global policies to tackle obesity share some similarities but also show differences depending on the specific circumstances and priorities of different countries and regions. The following are some of the main similarities and differences observed in the global policy response to obesity.

Similarly, global obesity policies include awareness and education. Most countries recognize the importance of raising awareness and educating people about obesity and its risk factors. This includes promoting healthy eating, physical activity, and the adoption of an overall healthy lifestyle [27]. For dietary guidelines, many countries, such as Europe, Asia and the Pacific, North America, and Latin America, have developed national dietary guidelines that provide recommendations for a balanced and nutritious diet. These guidelines often emphasize the consumption of fruits, vegetables, whole grains, and lean meats and limit the intake of added sugars, unhealthy fats, and processed foods [28]. What is more, promoting physical activity is also an important part. Encouraging regular physical activity is a common theme in global obesity policy. Countries work to create environments that support physical activity such as providing safe recreational spaces, promoting active transportation, and integrating physical activity into school and workplace settings [29]. Lastly, for food labeling and regulation, governments around the world, like the European Union, Russia, China, Brazil, and Australia, have implemented regulations to improve food labeling practices and make it easier for consumers to make informed choices [30]. This includes mandatory nutrition labeling, standardized pre-pack labeling, and restrictions on the marketing of unhealthy foods to children [31].

The differences in comparing these policies are, first, the specific focus of obesity policies may vary. Some countries like England, France, and New Zealand prioritize prevention efforts through school-based programs targeting children and adolescents while others like Mexico may focus more on interventions for adults or vulnerable populations such as low-income communities [32]. Also, different nations employ varying taxation and subsidy measures as strategies to combat obesity. For instance, certain countries impose taxes on sugary drinks and high-calorie products, whereas others provide subsidies for nutritious food items and use taxes and subsidies as a strategy to prevent obesity varies between countries [33]. The extent and effectiveness of these measures can vary depending on the local environment and political climate. What is more, for socio-economic factors, policies may need to address issues such as the affordability of food, the availability of resources for physical activity, and disparities in health services and education based on socio-economic status [34]. For cultural and dietary considerations, obesity policies need to take into account cultural differences and dietary preferences. Approaches to addressing obesity may vary in terms of the types of foods targeted, dietary patterns encouraged, and culturally appropriate messages. Lastly, the integration of obesity prevention and management into healthcare systems varies across the globe. Some countries like Germany and the United Kingdom have dedicated obesity clinics or specialist healthcare professionals, while others like Canada may rely more on primary care providers or community-based programs [35].

In addition, some countries like Chile, Canada, and Brazil have adopted pre-package nutrition labeling to help consumers make healthy choices [36]. Chile was the first country to implement warning labels for foods containing calories, sugar, saturated fat, or sodium above certain thresholds [36]. Similar warning labels are being introduced in Canada, Brazil, and Uruguay. In the United States, the revised Nutrition Facts table now includes an item for added sugars with a corresponding daily value percentage to help consumers meet their dietary goals [37]. Another major policy implemented is the elimination of artificial trans fats from the U.S. food supply. According to the WHO, trans fats are responsible for more than 500,000 premature deaths from cardiovascular disease worldwide each year. To address this problem, the WHO recently launched a campaign to replace trans fats in the global food supply by 2023 [38]. So far, the development of these policies has been successful in reducing obesity rates, which is still far from the target, but is a big step forward.

Addressing these challenges requires a multi-sectoral and collaborative approach involving policymakers, public health experts, healthcare professionals, educators, researchers, industry stakeholders, and community representatives. It is essential to prioritize evidence-based policies, invest in research, promote health literacy, and foster partnerships to tackle obesity comprehensively and effectively.

## Conclusions

It is important to note that these similarities and differences in various national policies to deal with obesity reflect the diverse nature of the global obesity epidemic and the need for tailored approaches to address it effectively. Collaboration and knowledge-sharing among countries can help identify best practices and inform the development of comprehensive and context-specific obesity policies. Obesity policy-making plays a critical role in improving nutritional status and health outcomes. For instance, obesity policies can focus on promoting healthy eating habits and addressing poor dietary patterns that contribute to obesity. Policies can encourage increased consumption of fruits, vegetables, whole grains, and lean proteins while reducing the intake of unhealthy foods high in added sugars, unhealthy fats, and sodium. By shaping the food environment, policies can help individuals make healthier choices and improve their nutritional status. Moreover, obesity is a major risk factor for various chronic conditions, including type 2 diabetes, cardiovascular diseases, certain types of cancer, and musculoskeletal disorders. Effective obesity policies can help prevent the onset of these diseases by addressing their underlying risk factors. By reducing obesity rates, policies can contribute to better health outcomes and lower healthcare costs associated with chronic disease management. Furthermore, obesity disproportionately affects certain population groups, including individuals with low socioeconomic status, racial and ethnic minorities, and marginalized communities. Obesity policies that focus on health equity can help reduce health disparities by addressing social determinants of health, promoting access to affordable and nutritious foods, and providing opportunities for physical activity in underserved communities.
